# Immediate development of post-varicocelectomy hydrocele: a case report and review of the literature

**DOI:** 10.1186/1752-1947-8-70

**Published:** 2014-02-26

**Authors:** Nader Salama, Saeed Blgozah

**Affiliations:** 1 Department of Urology, Alexandria Faculty of Medicine, Alexandria, Egypt; 2 Department of Urology, Hadramout Faculty of Medicine, Mukalla City, Yemen

**Keywords:** Varicocele, Hydrocele, Testicular lymphatics

## Abstract

**Introduction:**

Hydrocele development is the most common complication after varicocele repair. The beginning of this kind of hydrocele is variable. The shortest reported onset is one week. In the present report we describe an unusual immediate onset of hydrocele formation following varicocele repair. This represents the first report of a harsh hydrocele onset in the literature.

**Case presentation:**

A 29-year-old Caucasian male noticed the development of a big hydrocele just a few hours after inguinal varicocelectomy. The hydrocele remained stable in size throughout 12 years until a hydrocelectomy was done.

**Conclusion:**

Mass ligation and division of the spermatic cord structures during varicocele surgery should be avoided. Instead, lymphatic sparing is highly recommended. Differentiation between testicular edema and hydrocele should be confirmed as early as possible to assure the patient properly. This case study highlights the importance of our knowledge about the surgical anatomy of the spermatic cord structures. It definitely advances our understanding of a post-varicocelectomy hydrocele etiology and development. It is an original case report of interest to andrologists, urologists and general surgeons.

## Introduction

A hydrocele is an abnormal collection of fluid, usually serous, in the sac of the tunica vaginalis. It represents the most common complication after varicocele surgery [1-12]. Hydrocele formation following varicocele surgery or a post-varicocelectomy (PV) hydrocele has a lymphatic origin [5] due to iatrogenic disruption of lymphatics lying in and along the spermatic cord during varicocelectomy.

The onset of a PV hydrocele is an important point which may describe the natural history and pathogenesis of this hydrocele. Although PV hydroceles have been reported in many studies [1-12], several of these studies ignored their onset [12] while others showed a great variability in their timing [1-11]. Literature review showed that the fastest reported onset of a PV hydrocele was one month in adults [1] and one week in children [2]. Herein, we are reporting a case of PV hydrocele with extremely fast onset, highlighting how the surgical procedure originally used had several pitfalls resulting in the immediate development of this hydrocele.

## Case presentation

A 29-year-old Caucasian male physician consulted the Department of Urology for his varicocele problem with an abnormal spermiogram. His body weight and height were 79kg and 182cm, respectively. On physical examination, a grade III (GIII) (visible without Valsalva) left-sided varicocele was detected. Testicular volumes were 30mL on the right side and 22.5mL on the left side, as demonstrated by a Prader orchidometer. It was easy to notice a clear difference in the temperature on both sides of the scrotum during scrotal examination; the temperature of the left side was higher. This was also confirmed by the patient, who reported the existence of this temperature difference for many years. He was a single, non-smoker with no history of testicular pain even after a long day. In addition, he had no previous history of any form of scrotal trauma or epididymitis. He had no history of any medical troubles. His semen analysis revealed isolated asthenospermia with otherwise normal semen parameters. He expressed his wish to undergo varicocele repair as a remedy for his asthenospermia and to avoid any further deterioration to his semen parameters. He was also going to get married. Left non-microscopic inguinal varicocelectomy under general anaesthesia was performed by a senior staff member. Two young residents, one of them the first author of this manuscript, attended the surgery for support and training. The cord was identified, and a huge plexus of veins was easily seen even before opening the cord tunics. The vas complex was taken aside. Then, the cord was clamped with two artery forceps, and the segment in between was excised. Ligation of the two cord ends was performed. The testis was not delivered. The whole procedure was completed within 30 minutes with no blood loss.

About seven hours later, the patient left his bed to go to the toilet. Herein, he noticed an enlargement of the left side of his scrotum. He estimated this enlargement to be about three times the 30mL ball in the Prader orchidometer. The swelling was non-tense. He reported the event to the surgical staff on duty. They assured him that this enlargement was just scrotal edema after his surgery, although the scrotal skin could be pinched. They also added that this swelling would disappear within the next few days. He stayed in the ward overnight with an unremarkable course, and left for home the next morning. At home and 72 hours after the surgery, the scrotal enlargement remained the same size. He checked the enlargement himself using transillumination. Light shone through the enlargement and he realized that his swelling was a hydrocele.

**Figure 1:**
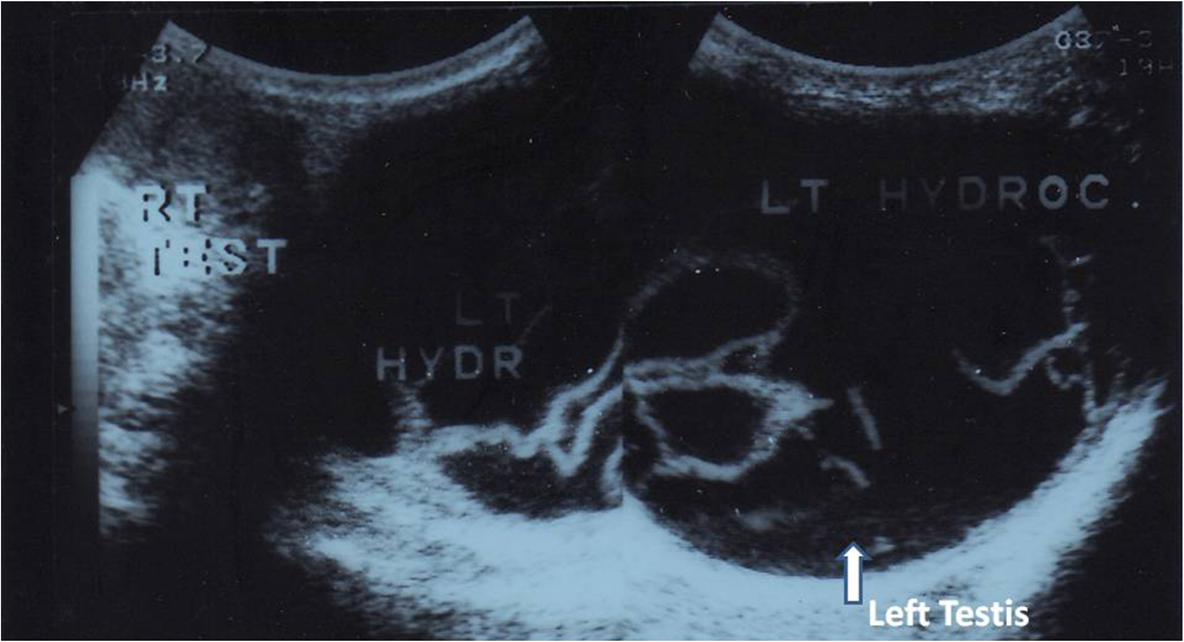
Scrotal ultrasound image showing multiple septa inside a large left-sided hydrocele sac, pushing the left testis inferiorly and laterally (arrow).

During the next years, he lived his daily life as usual. His sperm motility improved. However, the swelling remained the same size. It was always non-tense and painless. He did not try to receive any further treatment due to his first bad experience. Twelve years later, he decided to undergo hydrocelectomy for cosmetic reasons. He consulted us (the authors) with his hydrocele problem. A preoperative ultrasound evaluation showed a huge left-sided hydrocele with multiple internal septa (Figure 1) pushing the homo-lateral testis inferiorly and laterally. He underwent scrotal exploration and hydrocelectomy with excision-eversion of the tunica. The postoperative course was smooth and the pathology report of the tunical specimen was irrelevant. He has not reported any hydrocele recurrence for more than 12 years since this procedure. He never complained of any scrotal pain or discomfort during this period. He was satisfied with the cosmetic image of his scrotum.

## Discussion

Hydrocele development after varicocele repair represents the most common postoperative complication and ranges between 3 and 33% [1-12]. Understanding the natural history and pathogenesis of a PV hydrocele depends to a large extent on determining what caused the onset of the hydrocele. In our present report, the hydrocele formation was easily noticed by the patient himself as it reached a large size just a few hours following varicocele repair and before leaving the hospital.

In addition to the onset, special attention should be given to hydrocele size. Both onset and size of a PV hydrocele may represent a pivotal point in deciding the next and suitable treatment option. The treatment may range from “just wait and see”, to hydrocele tapping with or without injection of sclerosing agents, to surgical interference [1-12]. However, onset of hydroceles does not receive any attention in some studies [12] which mention nothing about it. In other studies, this onset had variable timing irrespective of the surgical procedure and the patient’s age with a mean ranging between 1 week to 22.1 months [1-11]. Interestingly, some of these studies reported an onset at 78 months [4] or even after 8 years following varicocele repair [1].

In our report, the reason for the development of the hydrocele could be attributed to the iatrogenic disruption of the testicular lymphatics during the varicocele surgery. This led to defective drainage of the fluid normally secreted by the tunica vaginalis into the lymphatics with its subsequent accumulation [5]. In support of this explanation, a number of factors were present. First, our patient had no past history of epididymitis or testicular trauma, which may act as precipitating factors triggering easy accumulation of hydrocele fluid after varicocele surgery [1]. Second, the excised tunica did not show any microscopic changes which might denote an underlying pathology. Third, ultrasound evaluation of our patient revealed the presence of multiple septa in his hydrocele sac. These sonographic features are characteristic of the protein content of the hydrocele [13]. This coincides with previous researchers who showed that PV hydroceles usually have a protein content [5].

The immediate onset of the hydrocele appearance in this reported case could be attributed to the total disruption of all testicular lymphatics going along the spermatic cord in our patient, who actually underwent clamping of all the cord structures except the vas complex. The surgeon’s negligence in not saving any of the testicular lymphatics was quite obvious during this varicocele surgery. Four lines of evidence were present to suggest this negligence. First, he did not use any optical magnification, at least not a surgical loupe which was available at the time the surgery was done. At present, surgeons use several techniques to preserve testicular lymphatics during varicocele repair. Examples of these include magnification during microscopic [8,9] or laparoscopic varicocelectomy [2,6] or usage of dyes such as isosulfan blue [7] to visualize the lymphatics. These measures can help in identifying the lymphatics and avoiding their disruption. Second, he did not try to follow what is highly recommended by many surgeons who always incorporate less surrounding tissue and remain as close as possible to the testicular vessels to avoid unnecessary injury to the cord lymphatics [9]. Third, he did not spare the internal spermatic artery, although it is well known that artery sparing varicocele surgery decreases the risk of hydrocele formation as the artery is usually surrounded with tiny lymphatics which may act as a possible path for drainage of the tunical fluid and may stop hydrocele development [2,6]. Fourth, he did not ligate the varicose veins at the highest point in the cord in order to leave the different lymphatic washout pathways [3]. This immediate development of a PV hydrocele in our patient exactly coincides with Zampiere *et al.*[3] who stated “. . . it is easy to understand that if hydrocele is caused by complete ligation of the lymphatic vessels and of alternative lymphatic washout pathways, the patient will suffer early onset of hydrocele with very small chances of spontaneous resolution or resolution with aspiration”. It is now well known that there are three main groups of lymphatics which emerge from the testis surface to go with testicular vessels along the spermatic cord. It is, therefore, highly recommended that every effort should be paid to save at least one group in order to give a chance for a reasonable lymphatic drainage of the testis [14].

This fast development of a PV hydrocele in our patient may also be related to some peculiar clinical findings in him. First, he had a GIII varicocele. Some surgeons [6] showed that PV hydrocele formation was more common in their patients with GIII varicocele (61.1%) than in those with GII varicocele (38.9%). So, is there any relation between the grade of varicocele and the number of accompanying lymphatics? Many researchers [10,11] state that there is a highly variable number of testicular lymphatics among men with varicoceles. Unfortunately, these researchers did not try to correlate between the number of demonstrated lymphatics and the grades of varicoceles in their studies. Second, the varicocele repair of our patient started with shifting the vas complex aside. This might compromise the perivasal lymphatics themselves [14]. Although they are few and very delicate, they still represent alternative pathways for testicular lymph flow [3]. Third, the lymphatic system in general is a highly complex system with highly inconsistent structures among different individuals [15]. This may entail a congenital lack of some alternative lymphatic washout pathways [3]. If this was the case in our patient, whose main testicular lymphatics have been iatrogenically crushed, then it would not be surprising to have the development of a sizable PV hydrocele. Our case represents the first documented instance of the immediate onset of hydrocele formation following varicocele repair, either in an adult or adolescent.

## Conclusion

Lymphatic sparing during varicocele surgery is highly recommended in order to avoid hydrocele development. The technique of complete spermatic cord ligation, therefore, should be considered obsolete during surgical intervention for a varicocele. An immediate increase in the scrotal size after varicocelectomy is not always testicular edema, which subsides with time, but can be a hydrocele of acute onset. Careful checking of the patient in such situations is of the utmost importance to accurately define the cause and, hence, assure that the patient is properly treated. Our case report highlights the importance of our vigilant understanding of the surgical anatomy of spermatic cord structures and their meticulous manipulation. It significantly expands our understanding of the PV hydrocele etiology and development. It is an original case report of interest to several specialties, including andrologists, urologists and general surgeons.

## Consent

Written informed consent was obtained from our patient for publication of this case report and any accompanying images. A copy of the written consent is available for review by the Editor-in-Chief of this journal.

## Abbreviations

G: Grade; PV: Post-varicocelectomy.

## Competing interests

The authors declare that they have no competing interests.

## Authors’ contributions

NS analyzed the data, collected the documents and followed-up with the patient. SB interpreted the patient’s data. Both authors contributed in writing and drafting the manuscript. They have read and approved the final manuscript.

## References

[B1] Amelar RD Early and late complications of inguinal varicocelectomy J Urol 2003 170 366 369 10.1097/01.ju.0000074975.79734.17 12853776

[B2] Esposito C Valla JS Najmaldin A Shier F Mattioli G Savanelli A Castagnetti M McKinley G Stayaert H Settimi A Jasonni V Guys JM Incidence and management of hydrocele following varicocele surgery in children J Urol 2004 171 1271 1273 10.1097/01.ju.0000112928.91319.fe 14767329

[B3] Zampieri N El-Dalati G Ottolenghi A Camoglio FS Percutaneous aspiration for hydroceles after varicocelectomy Urology 2009 74 1122 1124 10.1016/j.urology.2009.01.079 19647305

[B4] Misseri R Gershbein AB Horowitz M Glassberg KI The adolescent varicocele. II: the incidence of hydrocele and delayed recurrent varicocele after varicocelectomy in a long-term follow-up BJU Int 2001 87 494 498 10.1046/j.1464-410X.2001.00110.x 11298041

[B5] Szabo R Kessler R Hydrocele following internal spermatic vein ligation: a retrospective study and review of the literature J Urol 1984 132 924 925 6492283 10.1016/s0022-5347(17)49950-2

[B6] Hassan JM Adams MC Pope JC 4th Demarco RT Brock JW 3rd Hydrocele formation following laparoscopic varicocelectomy J Urol 2006 175 1076 1079 10.1016/S0022-5347(05)00402-7 16469622

[B7] Oswald J Körner I Riccabona M The use of isosulphan blue to identify lymphatic vessels in high retroperitoneal ligation of adolescent varicocele avoiding post-operative hydrocele BJU Int 2001 87 502 504 10.1046/j.1464-410X.2001.00171.x 11298043

[B8] Cayan S Acar D Ulger S Akbay E Adolescent varicocele repair: long-term results and comparison of surgical techniques according to optical magnification use in 100 cases at a single university hospital J Urol 2005 174 2003 2006 10.1097/01.ju.0000176488.44895.7b 16217378

[B9] Grober ED Chan PT Zini A Goldstein M Microsurgical treatment of persistent or recurrent varicocele Fertil Steril 2004 82 718 722 10.1016/j.fertnstert.2004.03.028 15374720

[B10] Libman JL Segal R Baazeem A Boman J Zini A Microanatomy of the left and right spermatic cords at subinguinal microsurgical varicocelectomy: comparative study of primary and redo repairs Urology 2010 75 1324 1327 10.1016/j.urology.2009.11.033 20188403

[B11] Hopps CV Lemer ML Schlegel PN Goldstein M Intraoperative varicocele anatomy: a microscopic study of the inguinal versus subinguinal approach J Urol 2003 170 2366 2370 10.1097/01.ju.0000097400.67715.f8 14634418

[B12] Dubin L Amelar RD Varicocelectomy: 986 cases in a twelve-year study Urology 1977 10 446 449 10.1016/0090-4295(77)90132-7 919135

[B13] Collings C Cronan JJ Grusmark J Diffuse echoes within a simple hydrocele: an imaging caveat J Ultrasound Med 1994 13 439 442 8083943 10.7863/jum.1994.13.6.439

[B14] Oswald J Brenner A Brenner E Fritsch H Schlenk B Radmayr C Pattern of lymphatic drainage of human testes with respect to hydrocele formation after varicocelectomy in adolescents [abstract] J Paediatr Urol 2009 5 S81

[B15] Foldi M Foldi E Kubik S Textbook of Lymphology for Physicians and Lymphedema Therapists 2003 Munich: Urban & Fischer

